# P382: Waste classification and management at King Abdulaziz Medical City in Jeddah (KAMC-J), Saudi Arabia

**DOI:** 10.1186/2047-2994-2-S1-P382

**Published:** 2013-06-20

**Authors:** N Nafouri, M Lamfon, B Al Subyei

**Affiliations:** 1 Infection Prevention and Control, King Abdulaziz Medical City - WR, National Guard, Jeddah, Saudi Arabia

## Introduction

Thousands of healthcare activities take place daily within KAMC facilities to improve health for patients which create wastes that must be carefully managed to mitigate environmental pollution.

## Objectives

The objective of this study was to assess the waste classification, the handling knowledge of generated waste and establish future needs.

## Methods

The authors surveyed 46 work areas’ supervisors via department heads using standardized form structured around the policy and targeted 46 clinical and non-clinical areas. Participants were asked to mark all applicable boxes.

## Results

A total of 40 surveys were analyzed with overall response rate of 87%. The authors looked at 10 different classes. The highest generated class was trash followed by sharps then biohazardous waste. When compared created waste to the functional activities carried in each area, 21 (53%) showed partial classification. Of 64 criteria, 34 (53%) recognized the proper utilization of the yellow bag for biohazardous waste. The red bag utilization for body parts was correctly marked as not applicable by the majority (93%). However, the theater where red bag is routinely used was not marked. The study showed common understanding of black bag usage for general trash except 9% miss use for other classes. Similarly, the usage of yellow container for sharps showed 70% proper utilization compared to 30% of improper use. An association was detected between the elevated number of blank and none answers on bags labeling indicating major procedure incompliance. The communication between the wastes collectors and the generating areas on discrepancies was found demanding by 93%. The labels for chemical and cytotoxic wastes were recognized as hard to obtain by 25%.

## Conclusion

The results suggest that the supervisors within the areas sampled, have knowledge of the waste hazards created in their areas and have been taking measures to control these hazards. Initiatives such as waste source reduction campaign at the point of generation so that most of the waste ends up in categories that can be reused, recycled, or that are safer and cheaper to dispose of help to raise awareness and support good control practice of waste management. Further studies on waste classification based on the functional activities became evident to meet new demands.

## Disclosure of interest

None declared

